# Pyorrhœa Alveolaris

**Published:** 1899-06

**Authors:** M. L. Rhein

**Affiliations:** New York, N. Y.


ARTICLE III.

### PYORRHEA ALVEOLARIS.

M. L. RHEIN, M. D., D. D. S., NEW YORK, N. Y.

Read before the Chicago Dental Society, Feb. 3, 1899.

Pyorrhea alveolaris describes a purulent discharge from the alveolar region. It is to-day the name most generally used to designate any form of pathological condition of the pericemental tissue, which, if neglected, after a certain lapse of time will be marked by more or less purulent discharges, and finally result in the loss of the teeth affected. It is impossible not to recognize the value of certain names that various men have endeavored to introduce as a substitute for the term pyorrhea alveolaris, because they all describe certain forms of this pathogenic condition very clearly. The fact that each of these names has its limitations to a few of the numerous types of this disease renders necessary a generic term. The name pyorrhea alveolaris is to day in use by a large majority as indicative of the entire field covered by the disease and can always be more clearly defined by the addition of proper adjectives where the etiology is understood.

The exciting cause of pyorrhea alveolaris is invariably some local irritation which, not being removed, sets up a condition of inflammation which prevents nutritive repair of the diseased pericemental tissue. This condition becoming permanent, results in the continued liquefaction of these tissues subject at a certain stage to infection, at which period the process of retrograde metamorphosis and consequent destruction of the entire pericemental tissue is more or less rapid according to the resistive energy of the individual's vitality.

The disease may be divided into two general classes: One where the exciting cause is practically the only cause of the disease, and which makes the condition purely a local trouble. This form may be called pyorrhea simplex in contradistinction to the other class, pyorrhea complex, where there is a predisposing cause back of the local irritant.

Pyorrhea simplex is a form of this disease to which it is my purpose to give very little attention this evening, though it is impossible to pass it by without some consideration. Having once begun to destroy the pericemental membrane, if allowed to progress without proper care and treatment, its results, as far as the loss of the alveolar parts are concerned, may be as destructive as that produced by any of the graver forms of this trouble. Pyorrhea simplex is seldom seen early in life except as the result of traumatism. Later in life lack of attention to the proper hygienic care of the mouth and the consequent encroachment of salivary calculi against the peridental membrane is perhaps the most common cause. "Dentistry up to date" as practiced by a large class of incompetent men bears the responsibility of being the cause of a large proportion of the cases of pyorrhea simplex. This may be illustrated more clearly by the mania which has overridden the profession for the past few years, and is happily now on the wane, for placing bridge work in every conceivable place. An ill fitting cap, the unnecessary use of ligatures, injudicious wedging, badly fitting plates, are a few instances of the frequent causes of attacks of local pyorrhea which may be laid at the door of the dentist himself. Similar attacks are also liable from any traumatic cause, such as a broken toothpick left between the teeth, a blow, pistol shot, a fractured tooth, etc. A great deal has been written at various times about the danger of pyorrhea infection extending from an affected tooth to others in the same mouth, but a close observation of many cases of local pyorrhea due to traumic origin, has clearly shown that the injured tooth or teeth are the only ones that are ever involved.

### PYORRHEA COMPLEX.

Although in all forms of this disease our chief duty is to keep the mouths of our patients free from any exciting causes and reduce all conditions of inflammation, still, where there is a predisposing cause which in itself is productive of the local inflammation and the irritation against which we are contending, this predisposing cause in such cases becomes *the* cause. In such cases all our efforts at purely local treatment are bound to fail in effecting any permanent results, unless this predisposing cause is either removed or checked. In fact, in these cases the local pathogenic manifestations known as pyorrhea alveolaris is only one of other results of this general interference with a proper equilibrium of the circulation. There is scarcely a vital disorder of any consequence which does not more or less interfere with the proper nutrition of the body. The extent of nutritional disturbance will invariably regulate the degree of severity of the local manifestation.

In order to properly appreciate this malady and to give the proper professional service to patients suffering from the disease, it becomes our first duty to discriminate as to the form of trouble which presents. A proper diagnosis between pyorrhea simplex and complex must first be settled before intelligent treatment can be commenced. It is unnecessary for me to enter into detail as to the manner of diagnosing a case of pyorrhea simplex by exclusion, except to say that every possible functional disturbance or toxæmia should be eliminated before any such diagnosis can be adopted.

The importance of a proper diagnosis can perhaps be more properly appreciated when we remember that our prognosis depends entirely upon the diagnosis which is made. To illustrate: A patient suffering in the last stages of diabetes or tuberculosis, etc., would present a very deplorable condition of the mouth. Having made our diagnosis, the prognosis would be so bad that no treatment other than the simple alleviation of local irritation would be attempted. No hope for any better result could be entertained. This is one of the many reasons why it is important that the forms of pyorrhea complex should be classified according to the predisposing disease in each case.

In August, 1894, at the meeting of the American Dental Association, held at Old Point Comfort, Virginia, I presented a method of classifying these forms of the disease which later has been practically followed out by Prof. W. D. Miller, of Berlin, in his "Text-book on Operative Dentistry." This classification is made by simply prefixing to pyorrhea an adjective stating the name of the disease which is causing the pathological symptoms in the oral cavity, as "gouty pyorrhea," "diabetic pyorrhea," etc. It is unnecessary to enumerate the subdivisions that might be listed, as they embrace all causes that may disarrange nutrition. In a paper such as this it is impossible to enter into the detail that is necessary in order to clearly appreciate each type of this disorder. There is comparatively as great a difference between tubercular pyorrhea and pregnancy pyorrhea as there is between tuberculosis and pregnancy. Almost as wide a difference in gouty pyorrhea from anæmia pyorrhea. All that can be done in this brief paper is to generalize and illustrate the differences that exist by citing some particular cases.

Most forms of the disease are accompanied by deposits of concretions upon the roots of the teeth of varying consistency and different compositions. These deposits vary greatly according to the form of the diseases; and again there are some forms where no deposits are found. Such a condition is found in liparous pyorrhea depending on a fatty infiltration of the pericardium, in which condition there is undoubtedly found more or less lipacidæmia. Such a condition can be perhaps better understood by giving history of a case from practice :

Mrs. S. presented in February, 1887, for treatment for "loose teeth." Married; age about thirty-five; childless. Local examination showed thirty-two teeth in normal position with perfect occlusion of all the teeth. No tooth had ever been filled, in fact, it was her first visit to a dentist since childhood. Except a small buccal cavity in the third right superior molar no defect was found upon any tooth. As far as lay in her power she had been scrupulously careful in reference to maintaining a cleanly condition of the teeth and mouth. No deposits of any kind could be found upon any of the teeth, nor upon the roots, although there were pyorrheal pockets extending half way up the roots on every tooth in her mouth. Slight pressure upon the gums would produce a whitish serous exudation from these pockets. She was very stout, in fact weighing over two hundred pounds, and was sent to me by her physician with the statement that she was under treatment for fatty degeneration about the heart This medical treatment at that time consisted mainly of long, rapid walks, early in the morning, with the free use of plenty of water and salines. In the oral cavity ordinary escherotic treatment, followed by stimulating and soothing applications was pursued. There followed a very marked improvement in the local symptoms. This was supplemented by instructing the patient in the proper manipulation of the brush so as to massage the gums over the roots as much as possible. Treatment was followed up at intervals for over a year, but without a thorough healing of the pockets, although their depths had been greatly reduced. In the spring of 1888 the patient went abroad and at Marienbad placed herself under general treatment. I did not see her again until 1890, when I found her weight very much reduced and she considered herself practically cured. All the local symptoms had entirely disappeared and no trace of any pyorrheal pockets could be found. A record of pyorrhea cured by constitutional improvement. I had an opportunity in the summer of 1898 of again examining the mouth of this patient and found everything in a normal, healthy condition.

One of the most remarkable characteristics of pyorrhea complex is the fact that it makes its appearance as one of the earliest symptoms of the disease of which it is a result. The other portions of the body which soon exhibit manifestations of malnutrition are the nails, the hair, the skin and the cornea of the eye, but none of them earlier than the pericemental tissue. In a paper entitled "Studies of Pyorrhea Alveolaris," published in the *Dental Cosmos* of March, 1888, I called attention to this fact by stating: "The gums and peridental membranes being fed by about the most remote portion of the blood tracts, it is no more than reasonable to suppose that these organs should be the first to exhibit symptoms of a lack of nourishment in their elemental corpuscles." This feature is a very valuable aid in diagnosing the true cause of this trouble, especially in the early stages when the general symptoms have not been very marked. It frequently falls to the duty of the conscientious dentist to be the first person to call the patient's attention to the beginning of some serious constitutional disorder. A careful observation will show clinical features in the local symptoms of Bright's pyorrhea that appear in no other form. By analogy, this undoubtedly holds good in every form of pyorrhea complex that exists and has been practically demonstrated by the writer in numerous cases. The unfortunate phase of this subject in our profession has been that definite authorities who believe in a constitutional cause of pyorrhea alveolaris have attempted to confine it to certain particular diseases.

Prof. Pierce in the extremest views which he gave forth as to uric acid being the cause of pyorrhea left open so many loopholes as to possibly obscure the serious role which uric acid plays in a large number of forms of pyorrhea. Uric acid—or, to be more exact, quadriurate—is a regular constituent of the blood. According to the latest researches it is safe to assert that uric acid is the result of the disintegration of the albuminous substances of the body, especially of the nucleins. Consequently, whatever should give rise to a greater form of metabolism, whether food, medicine, poison, or disease, would insure a greater amount of uric acid formation in the blood. As long as the kidneys perform their function in a normal, healthy way and carry off these products of metabolism disorders may be divided into two types: First, where there is an excessive formation of uric acid in the system as is found in gout. In these cases local disturbances are produced because all of this excess of uric acid is not at all times eliminated. When uncomplicated by disease of the kidney itself, general treatment is most efficacious. Second, where, through disease of the kidney itself, there is retention of uric acid in the system without necessarily any excessive quantity being formed. These are by far the graver forms for treatment and are the most difficult of diagnosis, because urinal examination demonstrate very little. It is unnecessary at this time to take any position as to how forms of these quadriurates reach the roots of the teeth, but the clinical fact remains that in certain cases of this class are met some of the most painful types of pyorrhea alveolaris and the most difficult of diagnosis, where no apparent lesion at the gingival border exisits, but where we find hard, flinty deposits near the apices of the roots. In these cases our chief reliance for diagnosis must depend not upon examinations of the urine, but upon examinations of the blood itself.

If theorizing is at all in order; I would quote from a paper of mine on the "Oral Expressions of Malnutrition," published in the *Dental Cosmos* of June, 1896: "Following along the line of Ebstein's theory, we can readily believe that on account of this deficiency of elemental corpuscles in the pericemental tissus a necrotic area is set up; a hyperacidity of the system being present at the same time, it follows that there is a strong tendency for the uric acid in the circulation to be deposited in this acid, narcotic area."

It is a well-known clinical fact that hyperacidity of the oral cavity is a marked feature of those forms of pyorrhea where serumal deposits predominate. This hyperacidity is unquestionably due to the lack of a proper alkalinity of the blood, and it has been thoroughly demonstrated that this lessening of the alkalinity of the blood favors the deposition of uric acid in the form of urates. There is no reason why this deposit should not occur on the roots of the teeth as well as in the phalangeal articutions. Assuming these facts to be as represented, there can be no question but that in all cases of functional disturbance, where, through disease of the kidney itself, elimination of uric acid is more or less incomplete, or in those cases where there is a large overproduction of uric acid, the urates become one of the important factors of local irritation and resulting inflammation.

One of the strongest opponents of Prof. Pierce's has been Dr. Talbot, of Chicago, who, if I have properly comprehended him, has studied the forms of neurotic pyorrhea and has endeavored to make some form of nervous disorder responsible for every type of this malady. Credit is due to Dr. Talbot for calling attention to the atheromatous condition of the capillaries in the pericemental region in severe types of pyorrhea. It is not my wish to underestimate the number of cases of pyorrhea alveolaris which owe their origin to nervous disturbances, but it is relatively hard to fix upon any exact ratio of the number of these as compared with other forms of pyorrhea. It is very likely that their number is greater than is generally believed.

While considerable attention has been given in these few remarks to the influence of vital disorders as a predisposing cause for various forms of pyorrhea, we should not lose sight of the important role played in this direction by poisons irrespective of whether the general condition is good or not. Mineral poisons sometimes administered with remedial intent, as mercury or bismuth, or the gradual absorption of the individual of some substance as lead, frequently is the direct cause of an attack. In this connection our attention may well be directed for a moment to a class of cases that are distinctly toxic pyorrhea and have frequently been very difficult to diagnose. In these cases there is found a lack of tonicity of the muscular coats of the intestines and considerable retention of fæces accompanied necessarily by absorption of more or less virulent toxines. My attention was first called to this condition from a case wherein the experience of clinical observation first led me to assume that the disease present was of the complex type; but the most careful medical examinations, repeatedly made, failed to find any distinctly functional disorder, until this clew was followed up. Treatment involving not only the toning up of the intestines but also rendering the contents thereof nontoxic, was followed by the most gratifying results. Since then, my attention has been directed to numerous instances of the absorption of intestinal toxines as the cause of pyorrhea alveolaris, and it is hoped that this attention drawn to the subject will be of some value to others in the treatment of some obscure cases.

Of all the forms of pyorrhea that have come under my notice in which the conditions, from a local standpoint, have been most serious, and where the prognosis is contradistinction has been most favorable, none have played so prominent a role as those owing their origin to some form of anæmia. It is remarkable how little attention has been given to this form of the disease and how common it is. It is frequently met as a plain, general anæmia, owing its origin to no particular source, and again we find the anæmic condition due to a functional disease of some special organ. The control of the general condition invariably brings about an easy cure of the local symptoms if too much of the pericemental attachment has not been destroyed. This condition is more frequently met among females and is often concomitant with uterine disorders. An illustration of such a case may be interesting: Miss F., spinster, age forty-nine, presented herself in December, 1896, for treatment for pyorrhea alveolaris which was manifest in the lower maxilla, the upper teeth with two exceptions being artificial. Pressure upon the gums produced the most copious discharge of pus from the socket of every lower tooth. Her dentist had informed her that all treatment for the preservation of these teeth would be useless, and that their loss was only a question of a short time. She came to me in order to find out whether I could give her any reasonable hope for the preservation of her teeth. The patient was thin, emaciated and careworn in appearance, and I was at once forcibly reminded of the anæmic condition present from the extreme pallor of the gums, lips and eyes. After a careful medical examination had been made the functional disturbance was narrowed down to uterine troubles and she placed herself under the care of Dr. H. N. Vineberg, who furnished me with the following resume of her condition and subsequent operation:

"January 13, 1897. Patient first consulted me. Has had uterine trouble for the past fifteen years. Has been treated from time to time, with temporary relief. In addition to pain in the right groin, back and down the right thigh, she suffers from frequent micturition. Examination disclosed the right kidney prolapsed to the line of the umbilicus. Uterus in retroversion of third degree. Right ovary prolapsed and enlarged to the size of an English walnut. The cervix hard, covered with erosions, and enlarged nabothian follicles. She was removed to a hospital and on February 3, 1897, a laparotomy was performed. Removed right ovary and tube. Ovary size of an English walnut and cystic throughout. Left tube and ovary apparently normal and left intact. Removed a small fibroid in anterior wall of the uterus. Sutured defect in wall of uterus with catgut. Ventrofixated uterus with two catgut sutures. Recovery uneventful, primary union of abdominal wound. March 6, uterus in good forward position, health excellent."

After the patient returned home in the spring of 1897 local treatment was instituted with the most satisfactory results. All concretions being removed from the roots, one application of a twenty-five per cent. solution of trichloracetic acid introduced into the pyorrheal pockets of not over two teeth at a time was sufficient for the thorough healing of each pyorrheal pocket. Since then the patient has given especial care to proper attention to hygienic measures, not only local but general; and as a result her general condition has visibly improved, so that she has lost the careworn expression and has gained materially in avoirdupois. There is a large quantity of salivary calculus that deposits on her teeth, and which has to be removed about every four months, but there has been no return of any distinctly pyorrheal condition.

A frequent complication of any form of pyorrhea alveolaris is an alveolar abscess. This is not only very prone to occur but is very difficult of diagnosis, and rarely suspected, because the purulent discharge from the abscess finds a direct vent through the pyorrheal pocket. On this account it is essential in the successful treatment of this trouble to be always assured that the vitality of the pulp is unimpaired, and, if there is any question as to its vitality, it is far better to remove a living pulp from such a tooth than to incur the risk of having the pus from an alveolar abscess constantly draining through a pyorrheal pocket, which, under such circumstances, you are ineffectually attempting to close up.

It has often been cited by advocates of the theory that all conditions of pyorrhea are purely local diseases; that the extraction of the teeth is sufficient to cure the disease. Never having heard this bold statement contradicted, I take this opportunity of stating that numerous cases of pyorrhea have come under my observation where the removal of the tooth in the diseased socket was not in itself sufficient to cure the disease in the socket.

It has been my aim to call your attention to the fact that, leaving pyorrhea simplex aside, the forms of pyorrhea complex that we may meet are only limited by the number of ills that humanity is heir to. Attention is also invited to the fact that each form of pyorrhea complex presents clinical symptoms so separate and distinct that they can easily be recognized by the observant dentist. His duty is to call the attention of his medical confreres to their diagnostic value in incipient functional disturbances.

Treatment: It is not my attention to consider extensively the details of local treatment, except to state some special forms of practice which have perhaps been distinctive with myself.

The necessity for a careful removal of every form of deposit, although disputed by a few, has been recognized to be of primary importance by the majority of men who treat this trouble. Your attention is called to the danger of handling too many teeth at one sitting and producing too much irritation instead of benefiting the patient. The form of escharotic used matters but little as long as the superficial tissues of the pocket are thoroughly removed and the parts stimulated without undue severity. They should always be left covered with some soothing application which should tend to keep the entrance to the pockets sealed for as long time as possible. A favorite application with myself has been the following prescription, which is not original:

| | | |
|---|---|---|
| Purified gum. lac. | - - | 135.0 |
| " benzoin | - - - | 5.0 |
| Carbol. acid cryst. | - - | 50.0 |
| Oil cinnamon, | - - - | 3.0 |
| Sacchartne, | - - - | 3.0 |
| Alcohol, | - | q. s. to make ½ litre. |

By allowing this to dry for a few moments it will be found to keep the pockets sealed for many hours as well as being beneficial from its therapeutic properties. So much has been written in reference to the nicety of detail as regards localized treatment that it is almost safe at the present day to say that the treatment of any individual case from a local standpoint must be left to the good judgment and sound common sense of the dentist who has the case in charge. An even occlusion of each and every tooth in the jaws is a *sine qua non* for success. The most difficult cases where other conditions have been made favorable are those where so much of the alveolar process has been lost that there is very little attachment left to keep the tooth in its socket. In many cases what attachment remains can frequently be improved by removing the pulp from the tooth by means of cocaine anæsthesia and thus diverting the portion of the circulation which has been nourishing the pulp to the pericemental tissues. This is, of course, only of value where there is a decided lack in the nutritional supply of the capillaries feeding the pericemental tissues; and nowhere does the good judgment of the operator come in more demand than when he determines that this course of procedure should or should not be pursued. Much has been written in the last few years about the value of splinting teeth which are too loose to stand unsupported in their own sockets. For temporary purposes ligatures, whether of silk, flax or metal, are valuable, but their durability is only a question of time, however skillfully they may be employed, and frequently much harm is done by the imperfect manner in which they are adjusted. No more injudicious treatment can be imagined than a practice which has been only too common—that of banding loose teeth together in order to obtain the benefits of this support. It is well nigh impossible to place bands around teeth without making them receptacles for the lodgment of debris and fermentable matter and consequently nests for the better breeding of the already too numerous bacteria. In a paper previously referred to, published in the *Dental Cosmos*, of March, 1888, I described a method of splinting teeth together by means of a combination of triangular wire lying in a groove cut in the occlusal portions of the teeth and held solidly in position by gold foil packed solidly under, around and on top of the wire, restoring in this manner the original conformation of the teeth except that the gold filling follows the triangular piece of iridio-platinum wire across the interspaces so that the teeth are solidly joined together at the occlusal angles. When a tooth is missing, or even a portion of a tooth, this can be attached to the wire before being permanently fixed in position.

While no false hopes of success should be held out to patients where the prognosis is of such a nature as to see the futility of much treatment, still, all our treatment is rendered unavailing unless considerable attention be given to the fact that constant co-operation is required from the patients themselves so far as the local treatment is concerned. They must be taught like children how to care for the oral cavity so as to insure the best hygienic condition attainable at all times. The same advice and teaching is not always applicable for all cases; and here again enters the question of the sound judgment and good advice of the dentist under whose treatment the teeth have been placed.—*Dental Review.*

